# Application of electrophysiological measures in degenerative cervical myelopathy

**DOI:** 10.3389/fcell.2022.834668

**Published:** 2022-08-09

**Authors:** Zhengran Yu, Wenxu Pan, Jiacheng Chen, Xinsheng Peng, Zemin Ling, Xuenong Zou

**Affiliations:** ^1^ Guangdong Provincial Key Laboratory of Orthopaedics and Traumatology, Department of Spine Surgery, The First Affiliated Hospital of Sun Yat-sen University, Guangzhou, China; ^2^ Department of Spine Surgery, Orthopedics Center of Guangdong Provincial People’s Hospital and Guangdong Academy of Medical Sciences, Guangzhou, China; ^3^ Department of Gastroenterology, Guangzhou Women and Children’s Medical Center, Jinan University, Guangzhou, China

**Keywords:** degenerative cervical myelopathy (DCM), electrophysiological studies, preoperative assessment, intraoperative monitoring (IOM), postoperative evaluation

## Abstract

Degenerative cervical myelopathy (DCM) is one of the leading causes of progressive spinal cord dysfunction in the elderly. Early diagnosis and treatment of DCM are essential to avoid permanent disability. The pathophysiology of DCM includes chronic ischemia, destruction of the blood–spinal cord barrier, demyelination, and neuronal apoptosis. Electrophysiological studies including electromyography (EMG), nerve conduction study (NCS), motor evoked potentials (MEPs) and somatosensory evoked potentials (SEPs) are useful in detecting the presymptomatic pathological changes of the spinal cord, and thus supplementing the early clinical and radiographic examinations in the management of DCM. Preoperatively, they are helpful in detecting DCM and ruling out other diseases, assessing the spinal cord compression level and severity, predicting short- and long-term prognosis, and thus deciding the treatment methods. Intra- and postoperatively, they are also useful in monitoring neurological function change during surgeries and disease progression during follow-up rehabilitation. Here, we reviewed articles from 1979 to 2021, and tried to provide a comprehensive, evidence-based review of electrophysiological examinations in DCM. With this review, we aim to equip spinal surgeons with the basic knowledge to diagnosis and treat DCM using ancillary electrophysiological tests.

## Introduction

Degenerative cervical myelopathy (DCM) is related to spinal cord neural dysfunctions caused by degeneration and acquired stenosis of the cervical functional spinal unit (FSU), which was comprised of the intervertebral disc, adjacent vertebra, endplate, facet joints, and paravertebral muscle together (Badhiwala et al., 2020). In normal conditions, the integrity of FSU maintains not only the spinal biomechanical steady and flexibility, but also protects and provides environment for neural tissue homeostasis inside the spinal canal. In DCM however, the degeneration of FSU such as cervical spondylosis, disc protrusion, or ossification of the posterior longitudinal ligament (OPLL) cause the cervical spinal cord compression and myelopathy (White and Panjabi, 1988; Baptiste and Fehlings, 2006).

The DCM diagnosis primarily depends on the clinical signs or symptoms suggesting involvement of spinal long tracts (spastic paraparesis associated with a variable degree of lower limb ataxia) and motor and sensory neurons in the gray matter (compromised sensory and motor function) (Mayfield, 1979). Neuroimagings including magnetic resonance imaging (MRI) of the spinal cord can show canal stenosis and signal abnormalities at the cervical cord lesion, but cannot directly indicate the neural dysfunction in DCM. Electrophysiological testing is thus recommended as an extension of the history, physical and radiographic examinations, for it can be used to assess the conductive functions of central and peripheral neural pathways. The value of electrophysiological examinations in the DCM assessment is multifaceted: 1) they help diagnosis and enable quantitative longitudinal assessment; 2) they help to rule out other neuromuscular diseases including peripheral neuropathy and motor neuron disease, which mimics DCM; 3) they can be used to predict the outcomes after decompressive surgeries (Dvorak et al., 2003; Capone et al., 2013; Nardone et al., 2016; Badhiwala et al., 2020).

**FIGURE 1:**
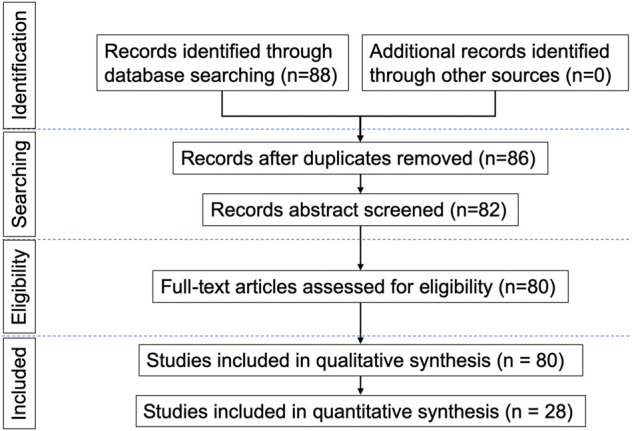
Flow chart illustrating the selection and inclusion process.

We reviewed and summarized published electrophysiological studies in DCM patients, in order to assess their indication and usefulness in this disease. The MEDLINE and EMBASE electronic databases were searched using the medical subject headings (MeSH): ‘compressive myelopathy’, ‘cervical spondylotic myelopathy’, ‘degenerative cervical myelopathy’, ‘neurophysiology’, ‘electrophysiology’, ‘transcranial magnetic stimulation’, ‘evoked potentials’, ‘electromyography’ and ‘nerve conduction studies’, and full-text articles in English language were retrieved. Both prospective and retrospective studies were included. Two reviewers evaluated the methodological quality of each study and risk of bias independently. The search strategy described above yielded 88 results. Only articles reporting data on studies using the above-mentioned neurophysiological techniques in patients with DCM were considered eligible for inclusion; therefore, 80 papers were provisionally selected and contributed to this review, among of which 28 papers were included in quantitative synthesis (meta-analysis). The earliest paper was published in 1979 and the most recent in 2021. A flow chart (Figure 1) illustrates the selection/inclusion process.

## Physiological basis and anatomical origins of spinal-cord-related electrophysiological tests

Evoked potentials (EPs) or evoked responses refer to the specific electrical activity generated by the nervous system (including peripheral or central) after receiving an internal or external stimulation. The neuronal membrane electrical activity underlies the generation and transduction of EPs. Action potentials in neuronal cell membranes can be generated in response to effective stimuli. In unmyelinated axon, the action potential is propagated because more voltage-gated Na + channels are opened as the depolarization spreads. As that depolarization spreads, new voltage-gated Na + channels open and more ions rush into the cell, spreading the depolarization farther along the length of the axon. In myelinated axons, electrical currents jump from one Ranvier node to the next, and the conduction velocity is significantly faster than that of unmyelinated axons. The conduction of evoked potential is also influenced by synaptic transmissions. By using these features and properties, researchers or clinicians can thus exert standardized artificial stimuli, such as electrical current, sound, light and magnetic field in a strictly controlled manner in respect to the quantity, intensity and frequency on the corresponding nervous structures to produce stable and reproducible EPs. Along with the standardization of recording and analysis methods, the electrophysiological tests can be used for mutual communication and clinical research applications.

**FIGURE 2:**
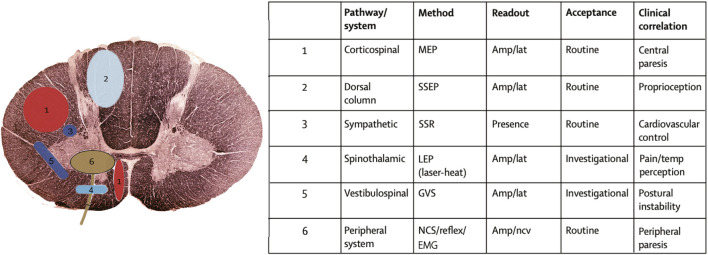
Neurophysiological techniques to study the function of specific spinal tracts and of the peripheral nervous system (adapted from (Dietz and Curt, 2006)). The clinical neurological examination can be complemented by electrophysiological recordings to obtain quantifiable measures about the affection of different spinal pathways. The location of the spinal pathways outlined in the table are numerically assigned in the schematic diagram. MEP = motor evoked potentials; SSEP = somatosensory evoked potentials; SSR = sympathetic skin response; LEP = laser evoked potentials; GVS = galvanic vestibular stimulation; NCS = nerve conduction study; EMG = electromyography; AMP = amplitude; LAT = latency; NCV = nerve conduction velocity.

Different electrophysiological tests have specific stimuli and recording methods, and thus have different use and values in the context of DCM management. Electromyography (EMG) is a test of muscles and also an indirectly test for nerve damage of the supplying motor nerve fibers. It is highly sensitive for detecting neuromuscular damage due to anterior horn cells destruction from compression and ischaemia in DCM, as well as differentiating DCM patients from patients with musculogenic lesions (Dvorak et al., 2003). The nerve conduction study (NCS) includes the compound muscle action potential (CMAP) and sensory nerve action potential (SNAP), which are used to assess the function of motor and sensory nerves, respectively (Tavee, 2019). The F wave and H-reflex are late CMAP examining the nerve roots conduction (Jerath and Kimura, 2019). The cutaneous silent period (CSP) is a robust and reproducible nociceptive EMG suppression, mediated by small-diameter A-δ afferents at the spinal level (Kofler et al., 2019). The MEPs are recorded over target muscles and are stimulated over the motor cortex and spinal roots with a transcranial magnetic (TMS) or electrical (TES) method (Lefaucheur, 2019). Robust normative MEP latency and conduction time variables can be established for healthy controls. The delayed central motor conduction time (CMCT) in DCM could be caused by several factors: slowed conduction in demyelinated corticospinal fibers, conduction along other oligosynaptic pathways, or reduction of size and synchrony of corticospinal volleys reaching the anterior horn cells (Felix and Wiesendanger, 1971). MEP amplitude may be unstable and no normative data can be reliably used in clinical practice. However, an obvious asymmetry in MEP morphology or size (>50%) is relevant for diagnosis (Lefaucheur, 2019). Somatosensory evoked potentials (SEPs) are time-locked electric potentials stimulated at the sensory peripheral nerves and recorded along the large-fiber somatosensory pathway. The SEPs mainly reflect the transduction functions of the dorsal column (Muzyka and Estephan, 2019). Contrary to SEPs, the laser evoked potentials (LEPs) and contact heat evoked potentials (CHEPs) are ascending sensory signals recorded from the scalp, but evoked by physical stimuli on dermatomes on the skin. Both the LEPs and CHEPs can be used to study spinothalamic tract conduction all along the spinal cord (Cruccu et al., 2000; Chen et al., 2001; Iannetti et al., 2001). (Figure 2 Adapted from (Dietz and Curt, 2006)) However, it is difficult for them to identify the precise level of the spinal cord lesion in DCM and traumatic spinal cord injury patients because of the Lissauer tract. Lissauer tract is a white matter tract in the spinal cord that projects up or down across one or two spinal segments. Somatosensory information arising from the skin must go through the Lissauer before entering into the dorsal horn of the spinal cord.

## Electrophysiology tests for DCM diagnosis

### Diagnostic sensitivity

“Diagnostic sensitivity” is the percentage of persons who have a given disorder (DCM) who are identified by the assay (Electrophysiological tests) as positive for the disorder. Detection of DCM is sometimes difficult, especially in those patients presenting without typical myelopathic signs or clinical and radiological mismatch. Objective measure of spinal cord dysfunction by MEPs and/or SEPs could help solve this problem (Abbruzzese et al., 1988; Maertens de Noordhout et al., 1991; Di Lazzaro et al., 1992; Herdmann et al., 1992; De Mattei et al., 1993; Tavy et al., 1994; Chistyakov et al., 1995; Kameyama et al., 1995; Chan et al., 2009). Table 1 summarizes the use of classical neurophysiological (MEPs, SEPs, EMG/NCS) tests for detecting preclinical, mild, and clinical DCM patients from various studies. The preclinical DCM, also called as presymptomatic or silent DCM, refers to patients with positive MRI signs but without any DCM symptoms (Bednarik et al., 2008). Mild DCM is defined as positive MRI signs with a modified Japanese Orthopaedic Association (mJOA) score >15 points, or with non-specific complaint of cervical pain, headache, dizziness, hand or leg paresthesia (Feng et al., 2020). Patients with clinical long-tract signs and symptoms, irrespective of the presence of MRI signs are classified as the clinical DCM patients (Lo et al., 2004). For these patients with unmatched clinical and radiographic presentations, electrophysiological tests were especially useful in confirming the diagnosis and predicting early progression (Bednarik et al., 2008).

**TABLE 1:** Diagnostic sensitivity of neurophysiological tests for preclinical or mild DCM.

References	NO. Patients	MEPs	SEPs	EMG/NCS
	30 preclinical	Abnormal MEPs(36.7%)	Abnormal SEPs (40%)	
	23 preclinical	Abnormal MEPs (65%)		
Tavy et al. (1999)	25 preclinical	Abnormal MEPs (8%), not significantly different from controls	Abnormal SEPs (4.3%), not significantly different from controls	
Bednarik et al. (2004)	66 preclinical	Abnormal MEPs (19.7%)	Abnormal SEPs (15.2%)	abnormal upper limb EMG (21.2%)
Bednarik et al. (2008)	199 preclinical	Abnormal MEPs (18.6%)	Abnormal SEPs (18.6%)	abnormal upper limb EMG (23.1%)
Masur et al. (1989)	15 preclinical, 4 DCM	Preclinical: Abnormal MEPs (86.7%); DCM: Abnormal MEPs (100%)	Preclinical: Abnormal SEPs (80%); DCM: Abnormal SEPs (100%)	
Kameyama et al. (1995)	24 preclinical, 67 DCM	Preclinical: no significantly different CMCT or silent period compared with normal control; DCM: significantly prolonged CMCT		Preclinical: no significantly different silent period compared with normal control; DCM: significantly shortened silent period
Simo et al. (2004)	29 preclinical, 22 DCM	Preclinical: abnormal MEPs (10%), DCM: abnormal MEPs (81.8%)	Preclinical: abnormal SEPs (7%), DCM: abnormal SEPs (45.5%)	
Kerkovsky et al. (2012)	27 preclinical, 18 DCM	Preclinical: abnormal MEPs (25.9%), DCM: abnormal MEPs (50%)	Preclinical: abnormal SEPs (29.6%), DCM: abnormal SEPs (55%)	
Nakai et al. (2008)	48 clinical DCM, 6 without MRI abnormality, 42 with positive MRI findings		Abnormal SEPs (90%) in all clinical DCM; DCM without MRI sign: abnormal SEPs (66.7%), DCM with MRI sign: abnormal SEPs (92.9%)	
Lo et al. (2004)	141 clinical DCM. 28 without MRI cord compression, 113 with mild to severe MRI cord impingement	DCM without MRI compression: abnormal MEPs (0%); DCM with MRI compression: abnormal MEPs (91.2%)		NCS and EMG showed changes supportive of radiculopathy (72%)
Lo et al. (2006)	223 clinical DCM. 50 without MRI cord compression, 176 with MRI cord impingement	DCM without MRI compression: abnormal MEPs (0%); DCM with MRI compression: abnormal MEPs (98%)		DCM without MRI compression: abnormal EMG (18%); DCM with MRI compression: abnormal EMG (88.1%)
Feng et al. (2020)	200 mild DCM		abnormal SEPs (66%)	
Nakamae et al. (2010)	482 milder (non-operative) DCM, 349 operative DCM	Non-operative group: abnormal MEPs (75%). Operative group: abnormal MEPs (100%)		
Stetkarova and Kofler, (2009)	21 mild DCM	Abnormal MEP (90.5%)	Abnormal SEPs (47.6%)	Abnormal CSP (81%), abnormal EMG (33.3%)s

Preclinical DCM: MRI, signs (cervical canal stenosis, cord impingement or cord compression) without clinical symptoms.

Mild DCM: MRI, signs with mJOA, score >15 or with main complaint of non-specific cervical pain, headache, dizziness, hand or leg paresthesia.

Clinical DCM: myelopathic signs and symptoms (including weakness or numbness in the upper and lower limbs, hyper-reflexia, clonus, positive Hoffman sign) with or without MRI, signs.

DCM: myelopathic signs and symptoms with MRI, signs.

In preclinical DCM, the incidence of abnormal MEPs varied greatly from 8% (not significantly different from control subjects) to 86% (Masur et al., 1989), SEPs from 4.3 to 80%, and EMG from 21.2 to 23.1% (Bednarik et al., 2008). Despite the divergence between studies, it can be concluded that neurophysiological studies could detect dysfunction of the spinal cord which may predate cervical myelopathy symptoms (Travlos et al., 1992). Preclinical DCM patients with abnormal MEPs and SEPs were significantly more likely to develop clinical symptoms and signs compared to patients with normal evoked potential tests (Bednarík et al., 1998). It should be noted that seven of the 15 presented studies reported poor sensitivity (<20%) of classical neurophysiological methods (particularly SEPs) to detect incipient DCM, i.e. preclinical and mild DCM patients. Recently, some other neurophysiological recording methods of spinothalamic pathways including the LEPs and CHEPs are also feasible and sensitive to the assessment of damage to central sensory nerve fibres (Kramer et al., 2012; Haefeli et al., 2013). CHEPs are reported to be more sensitive to damage than SEPs and enable primary assessment of individual cervical segments by testing along defined dermatomes in traumatic spinal cord injury cases (Jutzeler et al., 2016; Jutzeler et al., 2017). These novel neurophysiological methods are promising in improving the detection sensitivity of conduction fascicular damage in incipient DCM.

In clinically diagnosed DCM patients, both the MEPs (Lo et al., 2006) and SEPs (Berthier et al., 1996; Nakai et al., 2008) have been reported to be equally or even more sensitive over MRI or myelography. In clinical DCM patients, the occurrence of abnormal evoked potentials were significantly higher in MRI compressed group compared with non-compressed group (Lo et al., 2004; Lo et al., 2006; Nakai et al., 2008). The CMCT was the most important parameter in MEPs, followed by the cortical MEP latency and CMAP/MEP ratio (Kalupahana et al., 2008; Takahashi et al., 2008). For SEPs, the mostly used parameters are the latency and amplitude of N13 and N20, followed by the N9-N13 and N13-N20 intervals of the median and ulnar nerve (Nakai et al., 2008). The Right-Left differences of the same patients’ MEP or SEP parameters are also crucial in detecting abnormalities. DCM patients’ CMCT prolonged significantly at cervical extension or flexion positions compared with that at neutral, and thus the dynamic MEPs could also be used to increase the diagnostic sensitivity (Park et al., 2020). We also developed dynamic SEPs to achieve higher diagnostic sensitivity and specificity for DCM than ordinary SEPs or MEPs (Qi et al., 2020; Yu et al., 2020).

NCS including CSPs can also be used in the assessment of mild DCM (Stetkarova and Kofler, 2009). The CSP is a protective reflex that is mediated by spinal inhibitory circuits and is reinforced in part by parallel modulation of the motor cortex, and abnormal CSPs are highly related to cervical intramedullary lesions and spinothalamic dysfunction (Kofler et al., 2003). Lo et al. (2007) reported abnormal CSPs in 96% of patients with the clinical diagnosis of DCM. Another study reported CSP abnormalities were more sensitive than SEP, almost equally sensitive as upper limb MEPs in detecting the DCM patients, but were highly associated with spinothalamic dysfunction (Stetkarova and Kofler, 2009).

### Diagnostic specificity (differential diagnosis)

“Diagnostic specificity” is the percentage of persons who don’t have DCM and are identified by the assay (Electrophysiological tests) as negative. There are many neurological conditions such as those caused by autoimmune, infectious, inflammatory, and metabolic abnormalities can present similarly to DCM, especially in cases where spondylosis may be coexistent. Excluding the coexistence of DCM is necessary for deciding the management methods. In this review we mainly discuss the differential diagnosis of DCM from multiple sclerosis (MS), amyotrophic lateral sclerosis (ALS), Hirayama disease (HD), cervical spondylotic amyotrophy (CSA) and peripheral nerve entrapment by using neurophysiological examinations (Table 2).

**TABLE 2:** Differential diagnosis.

	MEP	SEP	EMG/NCS/Others
DCM	Prolonged CMCT; significantly prolonged CMCT at flexion and extension neck positions	Decreased amplitude, prolonged latency; significantly decreased N13 amplitude at dynamic neck positions	NCS can be normal or can see signs of radiculopathy: prolonged F-wave, delayed CSP; EMG: can have long duration, high amplitude, polyphasic motor units with reduced recruitment
MS	Prolonged CMCT	Scalp-recorded SEPs are present in only 50–86% and short-latency N13 from the neck or P14 from the scalp in 69–94%	Abnormal visual-evoked potentials; May have abnormal brain auditory-evoked potentials
ALS	Normal or marginally prolonged CMCT, reduced MEP amplitude and abnormal morphology, reduced cortical threshold	Absent or significantly altered	NCS: CMAP reduced amplitudes of APB, ADM and FDI, especially APB. Higher UM ratio and lower SHI. Delayed CSP. EMG: Fibrillation and fasciculations
HD	Prolonged CMCT, especially upon flexion	decreased N13 amplitude and prolonged N13–N20 interval upon flexion	NCS: significantly lower ulnar CMAP amplitudes; lower U/M CMAP ratio
CSA	Prolonged CMCT	not change at dynamic neck positions	NCS: Slightly decreased ulnar and median CMAP amplitudes; normal U/M CMAP ratio
Peripheral nerve entrapment	Normal CMCT, prolonged PMCT	Abnormal Erb potential	Abnormal nerve conduction Velocity

MS patients usually affect a specific population (females age 20–40s) and most often have a history of visual symptoms and MRI periventricular white matter lesions, which can distinguish from DCM pathology (Kim et al., 2013). MEP, SEP or EMG parameters alone are not helpful in distinguish MS from DCM, but additional visual and auditory evoked potentials are useful as the MS also frequently affects the optic and auditory nerves (Hardmeier and Fuhr, 2021).

The ALS can be harder to differentiate from DCM as it presents weakness, muscle atrophy, fasciculations, gait difficulty, and no specific MRI features in the cervical spine. In ALS, the pattern of MEP abnormalities is different from that in DCM: the CMCT is usually reported as normal or marginally prolonged, with a reduced MEP amplitude and abnormal morphology in ALS patients (Eisen et al., 1993; Khalili-Ardali et al., 2021). This implies that in an ALS patient with radiological evidence of cervical spondylosis and/or myelopathy, a normal CMCT and normal or reduced threshold would suggest that the spondylosis and/or myelopathy is of no clinical relevance, and thus these patients should not be selected for surgical treatment. ALS patients’ SEPs were absent or significantly altered, which is not significant in differentiating them from DCM (Khalili-Ardali et al., 2021). NCS and EMG are of great significance in deciphering ALS from DCM. Kalita et al. (2017) introduced the split hand index (SHI) calculated by the CMAP of APB and ADM muscles, and found that a lower SHI was sensitive for screening ALS. Furthermore, EMG will demonstrate findings in all four limbs as well as the sternocleidomastoid, whereas patients with DCM will not exhibit abnormalities in the sternocleidomastoid (Kang and Fan, 1995; Ishpekova and Milanov, 2000).

HD and CSA are two kinds of disease characterized with weakness and wasting of upper limb muscles. In HD, dynamic neurophysiological tests show a reversible significant prolonged CMCT in MEP and decreased SEP N13 amplitude and prolonged N13–N20 interval only upon neck flexion (Restuccia et al., 2003; Zheng et al., 2017; Park et al., 2019), whereas DCM patients show MEP and SEP deterioration at both extension and flexion (Park et al., 2020; Qi et al., 2020; Yu et al., 2020). Furthermore, Preethish-Kumar et al. (2016) used EMG to reveal grossly reduced CMAP amplitudes of affected muscles in HD patients. CSA usually presents prolonged CMCT (Zheng et al., 2019), which is not helpful in the differential diagnosis of DCM. Dynamic SEPs are effective for differentiating DCM from CSA, for SEP amplitudes changed more significantly in DCM than CSA (Qi et al., 2020). Jin et al. introduced the use of CMAP and SNAP to differentiate CSA from ALS and HD (Jin et al., 2014). The ulnar/median CMAP ratio (UM ratio) was found to be significantly lower in HD, significantly higher in ALS and no different in CSA compared with the normal range from previous studies (0.89–1.60) and with the healthy controls (1.15 ± 0.23), indicating its value in the differential diagnosis of these diseases.

## Preoperative evaluations for DCM severity and characteristic

### Severity assessments

Some investigators also tried to correlate neurophysiological findings with clinical and radiographic signs quantitatively. CMCT is significantly related to disability measured by JOA score and clinical signs of hyperreflexia and the presence of a Babinski sign (Tavy et al., 1994; Kameyama et al., 1995). It is also correlated with MRI findings including the number of compression levels (Chistyakov et al., 1995), spinal cord compression degrees and intramedullary hyperintensity (Tavy et al., 1994; Misra and Kalita, 1998; Lo et al., 2004; Lo et al., 2006). SEPs prolonged latencies and decreased amplitudes also strongly correlate with clinical signs such as gait disturbance (Lee et al., 2011), the severity of myelopathy indicated by preoperative JOA scores, and MRI signs of spinal cord impingement and canal stenosis level (Restuccia et al., 1994; Lyu et al., 2004; Hu et al., 2008; Kerkovsky et al., 2012). Dynamic SEPs N13 amplitude ratio is associated with pre- and post-operative mJOA scores and several MRI measurements, demonstrating its role in evaluating disease severity and predicting postoperative prognosis (Yu et al., 2020). Chistyakov et al. (1995) used the combined test of MEPs, SEPs and F-wave responses and found that the central sensory and motor conduction time in DCM group was significantly prolonged, especially in patients with multiple stenotic segments compared to those with single disc herniation. Contrary to the SEPs results, the MEPs combined with F-wave results of patients with radiculopathy showed significant damage to peripheral conduction. Therefore, the combination of MEP and F-wave examination is more suitable for the evaluation of patients with radiculopathy, while the severity of conduction damage in myelopathic patients should be evaluated by the combination of MEPs and SEPs tests (Chistyakov et al., 1995).

Quantitative EMG examination reveals subclinical disorders of motor neurons even in patients with normal muscle power on manual testing, rather than long tract lesions in the spinal cord. It provides important perspectives on the status of muscle and motor neurons in DCM patients. Upper limb EMG motor unit potentials (MUPs) were related to radiologic level of cord compression and compression degree in DCM (Hattori et al., 2010). In DCM cases, increased mean duration of MUPs could result from axonal degeneration, denervation of the muscle fibers after partial loss of their motor neurons or axons, and reinnervation of the denervated muscle fibers by sprouting from adjacent terminal nerve branches (Hattori et al., 2010). Lower limb EMG is used to record muscle activity in order to analyze gait and functional balance in DCM patients, which enables care practitioners to objectively quantify disease severity and objectively documenting the effectiveness of their intervention, and may also lead to the development of new rehabilitation strategies (Haddas et al., 2018; Haddas et al., 2019). NCS including CSPs can be used in the assessment of mild DCM (Stetkarova and Kofler, 2009). The CSP onset latency was correlated with upper limb MEP CMCT, JOA score, and SEP N13 amplitude in DCM patients (Stetkarova and Kofler, 2009).

Above all, electrophysiological studies provide objective evidence for functional deficit in DCM patients, which are correlated with clinical manifestations, as well as spinal cord compression degree and locations in MRI imaging.

### Outcomes prediction

Although the effect of surgery in DCM seems to be beneficial, the prognosis varies among individuals (Braakman, 1994). Preoperative neurophysiological evaluation provides an effective tool for predicting postoperative prognosis, and thus could influence the decision on surgeries. Table 3 summarizes the prognosis prediction by neurophysiological tests. Multiple studies reported preoperative MEPs or SEPs are significantly correlated with both the preoperative and postoperative JOA score (Hu et al., 2008; Takahashi et al., 2008; Nakanishi et al., 2014; Feng et al., 2020). Prolonged MEP CMCT in DCM patients might suggest slowed conduction in demyelinated corticospinal fibers, conduction along other oligosynaptic pathways, or reduction of size and synchrony of corticospinal volleys reaching the anterior horn cells, usually associating with worse prognosis (Felix and Wiesendanger, 1971). In a comparison between CMCT data and surgical outcome, Yonenobu et al. (1986) reported poor prognosis in patients with prolonged preoperative CMCT and with enhancement of intensity in spinal compressed region in T2 contrast MRI image, because of the irreversible changes in spinal cord. For SEPs, N9-20 was most correlated with surgical outcomes among median SEP variables (Lyu et al., 2004). The trial-to-trial variability in SEP was reported to possess higher prognostic accuracy and sensitivity than the conventional averaged SEP (Cui et al., 2015). The N13 amplitude ratios in dynamic SEPs also significantly correlate with the baseline mJOA score and 2-years post-operative recovery ratio (Yu et al., 2020). A large-scale prospective study concluded that the presence of symptomatic cervical radiculopathy and electrophysiological abnormalities of cervical cord dysfunction detected by MEPs or SEPs were associated with time-to-DCM development and early development (<12 months) of DCM, while MRI hyperintensity predicted later (<12 months) progression to symptomatic DCM in pre-symptomatic (preclinical) patients (Bednarik et al., 2008). Thus, neurophysiological abnormalities might be an indicator for early surgical decompression in preclinical DCM patients. Combined use of SSEPs and MEPs can be helpful in evaluating patients with asymptomatic (preclinical) degenerative cervical spinal cord compression, as they can detect subclinical involvement of the spinal cord or nerve roots more sensitively than using either of them alone, thereby identifying patients who should be monitored vigilantly for development of myelopathy (Bednarík et al., 1998; Tavy et al., 1999; Bednarik et al., 2008; Wilson et al., 2013).

**TABLE 3:** Prognosis prediction by electrophysiological test.

References	NO. Patients	Follow-up	Electrophysiological test
	30 preclinical Conservative	2-years	1/3 patients with entry MEP or SEP abnormality (5 in 15) in comparison with no patients with normal EP tests (0 in 15) developed clinical myelopathic signs
Feng et al. (2020)	200 Conservative	1-year	SEP classifications predict decline in mJOA
Hu et al. (2008)	76 Surgery	1, 3, 6, 12, and 24 months	SEP classifications predict JOA recovery ratio
Yu et al. (2020)	39 Surgery	2-years	Dynamic SEP N13 amplitude ratios correlate with baseline mJOA score and 2-years post-operative recovery ratio
de Noordhout et al. (1998)	43 surgery; 12 conservative	1-year	MEPs: 10 in 43 normalized after surgery; 4 in 12 worsened without surgery
			SEPs: 5 normalized after surgery; 4 in 12 worsened without surgery
Misra and Kalita, (1998)	20 Conservative	1.5 and 3 months	MEPs: 15 in 20 improved at 1.5m, 4 in the above 15 further improved at 3 m
			SEPs: 11 in 20 improved at 1.5m, no change at 3 m
	30 conservative	6,12,24 months	The association between initial MEP or SEP abnormality and clinical manifestation of SCM during the 2-year period was statistically significant (Fisher’s exact test, *p* = 0.02)
	30 Surgery	6,12,24 months	MEP latency, amplitude and spinal cord motor conduction velocity (SCMCV) improvement after surgical treatment might occur in clinically milder patients but not in severe patients after 6 months. A lower SCMCV measurement in clinically severe patients may accompany an insufficient outcome of decompression surgery. Limited electrophysiological and neurologic improvement appears to occur at 1 or 2 years after surgery
Bednarik et al. (2004)	66 Conservative	≥2 years	13 patients with abnormal initial MEPs (19.7%): 5 developed myelopathy (38.5%) and 8 didn’t (15%); no significant difference
			10 patients (15.2%) with abnormal initial SEPs: 5 developed myelopathy (38.5%) and 5 didn’t (9.4%); the difference was significant (*p* = 0.016)
			14 patients with abnormal initial EMG (21.2%): 8 developed myelopathy (61.5%) and 6 didn’t (11.3%); difference was highly significant (*p* < 0.001)
Bednarik et al. (2008)	199 conservative	≥2 years	37 patients (18.6%) with abnormal initial MEPs: 18 developed myelopathy (40%) and 19 didn’t (12.4%); the difference was significant (*p* < 0.001); significantly related to early clinically myelopathy symptom (<12 months). 37 patients (18.6%) with abnormal initial SEPs: 17 developed myelopathy (37.8%) and 20 didn’t (13%); the difference was significant (*p* < 0.001); significantly related to early clinically myelopathy symptom (<12 months). 46 patients (23.1%) with abnormal initial EMG: 19 developed myelopathy (42.2%) and 27 didn’t (17.5%); the difference was significant (*p* < 0.001); significantly related to early clinically myelopathy symptom (<12 months)
Takahashi et al. (2008)	56 Surgery	1-year	CMCT for patients with poor outcome was significantly longer; CMCT of 15 milliseconds or more in the upper extremities or that of 22 milliseconds or more in the lower extremities indicated poor prognosis
Nakanishi et al. (2014)	42 Surgery	1-year	MEP latencies and CMCT were significantly shorter 1-year after surgery; The CMCT parameters before or 1 year after surgery correlated significantly with the JOA score both before and 1 year after surgery; CMCT recovery ratio from the longer CMCT in the ADM correlated significantly with the JOA recovery ratio
Tadokoro et al. (2019)	16 Surgery	3, 6, 12 months	Preoperative CSP abnormalities (84%). Preoperative and 1-year post-operative JOA scores did not vary significantly among CSP classification groups, probably because of the small sample size

Abnormal upper limb EMGs are also unfavorable predictors for DCM prognosis (Bednarik et al., 2004; Bednarik et al., 2008). Abnormal NCS and EMG can indicate anterior horn cell lesion in cervical cord and are associated with poor prognosis (Bednarik et al., 2008). The hindered upper or lower extremity EMG combined with T2WI intramedullary hyperintensity correlated with a worse post-operative recovery (Liu et al., 2013).

## Intra-operative monitoring

Neuronavigation systems including the intraoperative CT, MRI and ultrasound techniques (Ganau et al., 2018a) as well as intraoperative neurophysiological monitoring (IONM) system including MEPs, SEPs and EMG are two kinds of technological aids routinely used high-risk spinal cord surgeries (Hilibrand et al., 2004; Devlin et al., 2006; Clark et al., 2013; Hadley et al., 2017; Takeda et al., 2018). The former is mainly for guiding the surgical team step by step and the latter is mainly for detecting changes in spinal cord function related to patient pre- and intra-operative positioning, hemodynamic effects during anterior cervical discectomy and fusion, and C5 injury during posterior laminectomy (Bose et al., 2007; Wang et al., 2016; Wang et al., 2020). The utilization of multimodal IONM can assist the surgeon in taking corrective measures to reduce or prevent permanent neurological deficits, and thus minimize the occurrence of position-related brachial plexus injury, post-operative C5 palsy, paraparesis and other complications in both anterior and posterior approach surgeries (Fan et al., 2002; Bose et al., 2007; Jahangiri et al., 2011; Clark et al., 2013). Intraoperative MEP is generally reckoned as the most important monitoring method, and is most related to post-operative prognosis (Hilibrand et al., 2004; Clark et al., 2013; Wang et al., 2020). Wang et al. reported some patients could have intraoperative MEP improvement after the procedure of cervical cord decompression, and these patients showed a better immediate and long-term neurologic recovery compared with those without intraoperative MEP improvement (Wang et al., 2016). Another study reported that positive changes in MEP during IONM may affect functional improvement 1 month after operation and early discharge without significant complications in DCM patients (Park et al., 2018). Several mechanisms can explain the relationship between intraoperative MEP and postoperative functional improvement. One is that in DCM, nervous tissue of the spinal cord does not undergo necrosis but limits the capability of neurological function; thus, it is reversible through surgical decompression (Wang et al., 2016). Thus, improvements in MEP after neural decompression are probably due to improvements in the excitability of neurons or the corticospinal tract (Macdonald, 2006). Secondly, an increase arterial supply can also alleviate spinal cord ischemia and thus result in MEP improvements during surgery (Wang et al., 2016).

Intraoperative SEP alerts also had a high sensitivity and specificity for predicting new neurologic deficits in the early postoperative period (Garcia et al., 2010), and the use of SEPs to monitor upper extremity nerves before and during surgery also a valid and useful technique to minimize the brachial plexus injuries during positioning and surgical procedures (Jahangiri et al., 2011; Plata Bello et al., 2015). The dorsal column function indicated by SEPs might be more vulnerable to the compression, and thus, the lack of significant changes in SEP after cervical decompression might be related to the anatomical vulnerability of this region. Previous studies also revealed that MEP changes are more sensitive than SEP changes during surgery (Hadley et al., 2017).

## Post-operative evaluation for DCM patients

Functional improvement as indicated by symptomatic relieve and increased clinical assessment scores such as JOA and mJOA after decompression surgery are well recognized. However, these assessments are usually subjective and cannot directly reflex the neural conductive function. Postoperative electrophysiological studies may provide valuable information in quantifying the degree of functional involvement of the spinal cord after surgery. Pre- and 1-year post-operative MEP tests indicate that cervical laminoplasty improves corticospinal tract function as presented by shortened CMCT (Nakanishi et al., 2014). Further, the CMCT parameters before or 1 year after surgery correlated significantly with the JOA score both before and 1 year after surgery, and the CMCT recovery ratio from the longer CMCT in the ADM significantly correlated with the clinical recovery ratio (Nakanishi et al., 2014). de Noordhout et al. (1998) tracked the MEP and SEP changes in DCM patients who received either surgeries or conservative treatments in a 1-year period. They reported that in surgically treated DCM patients, MEP abnormality changed from 95.3 to 72.1%, while from 66.7 to 91.7% in conservatively treated patients in 1 year. The tibial SEP and MEP abnormalities persisted in spite of clinical improvement in most surgically treated patients, which probably reflects permanent vascular or necrotic lesions induced in the cord by spondylotic changes. Some authors also reported discrepancy between functional recovery and electrophysiological findings in DCM patients after surgery. Tadokoro et al. (2019) reported CSP abnormalities persisted after surgery in most cases in a 1-year period, indicating irreversible damage of the intramedullary reflex circuit, despite the JOA score recovery. The high sensitivity of neurophysiological studies including MEPs, SEPs and NCS might make them useful to monitor disease progression in post- or unoperated patients. The phenomenon of JOA score recovery without neurophysiological recovery also provides insight into postoperative neural recovery in DCM.

## Conclusion and future prospects

In conclusion, the clinical and radiographic presentations of DCM are highly variable, making the diagnosis difficult in some cases. Electrophysiological studies exhibit an excellent sensitivity in identifying spinal neural compromise, but are of less value in the differential diagnosis, which can be improved by using the dynamic SEPs and MEPs. Neurophysiological tests are useful for assessing cervical cord dysfunction and predicting the prognosis of DCM, and thus are valuable in deciding the treatment methods. They are also useful in monitoring neurological function during surgeries and disease progression in post- or unoperated patients during follow-up rehabilitation.

For future perspectives, machine-learning and artificial intelligence are warranted to decipher more information from those multi-dimensional neurophysiological results. Hu et al. (Zhang et al., 2009; Cui et al., 2015; Wang et al., 2017; Cui et al., 2019) used the random forests-based time-frequency analysis technique to sort out meaningful information contained in various SEP components, in order to identify lesion locations, quantify the severity and predict prognosis in both spinal cord compression rat models and DCM patients. However, these studies contained only a relatively small sample, and more clinical studies are required to assess the validity of this technique in humans. Moreover, machine learning-based neurophysiological studies could be used to detect neurological deficits and predicting response to various treatment of DCM more precisely in the future. Along with the relevance of electrophysiological measures at various timepoints in the management of DCM patients, other recent trends in basic and clinical research point toward the relevance of fast-paced advances in imaging, clinical diagnostic tools, molecular genetics, surgical techniques, and reparative/regenerative strategies (Ganau et al., 2018b). Altogether those research efforts are allowing spine surgeons to reshape the management strategies available for an aging population that suffers increasingly from this degenerative condition.
